# Evaluation of adipose-derived stem cells (ASCS) exosome implantation and platelet-rich fibrin (PRF) on critical long bone defects in Sprague-Dawley rats

**DOI:** 10.1007/s00590-024-03964-0

**Published:** 2024-05-23

**Authors:** Fahresa Hilmy, Ismail Hadisoebroto Dilogo, Mirta Hediyati Reksodiputro, Radiana Dhewayani Antarianto, Muslich Idris Al Mashur, Kevin Jonathan Adhimulia

**Affiliations:** 1 Department of Orthopaedic and Traumatology, Cipto Mangunkusumo General Hospital, Faculty of Medicine, Universitas Indonesia, Jakarta, 10430 Indonesia; 2 Department of Ear, Nose and Throat Clinic, Cipto Mangunkusumo General Hospital, Faculty of Medicine, Universitas Indonesia, Jakarta, 10430 Indonesia; 3 Department of Histology, Faculty of Medicine, Universitas Indonesia, Jakarta, 10430 Indonesia

**Keywords:** Adipose-derived stem cells (ASCs) exosome, Critical long bone defect, Platelet-rich fibrin (PRF), Sprague-Dawley

## Abstract

**Introduction:**

This study aimed to assess the efficacy of adipose-derived mesenchymal stem cell exosomes (ASCs exosome) and platelet-rich fibrin (PRF) in treating critical long bone defects in Sprague-Dawley rats. Critical long bone defects, defined as exceeding 2 cm or 50% of the bone diameter, often pose a healing challenge. While autologous bone grafts have been considered, they have shown unreliable results and donor-site complications, necessitating alternative treatments.

**Methods:**

The research followed a quasi-experimental post-test only control group design involving 30 male Sprague-Dawley rats. The rats were divided into five groups and subjected to femur bone defect creation, internally fixed with a 1.4 mm K-wire, and treated with various combinations of hydroxyapatite (HA), bone graft (BG), ASCs exosome, and PRF. Histomorphometry and BMP-2 gene expression analysis were performed to evaluate bone healing.

**Results and Discussion:**

The results indicated that the group treated with HA + BG + ASCs exosome (group IV) exhibited the highest BMP-2 gene expression, while group III (HA + BG + ASCs exosome + PRF) had the highest chordin level. Overall, groups receiving ASCs exosome or PRF intervention showed elevated BMP-2 expression compared to the control group. The use of ASCs exosome and PRF showed comparable outcomes compared to bone graft administration in terms of histomorphometry analysis.

**Conclusion:**

The administration of adipose tissue derived mesenchymal stem cells and PRF has a comparable outcome with the use of bone graft in terms of osseus area and expression of BMP-2 in critical bone defect.

## Introduction

Extensive bone defects can result from a variety of conditions, including trauma, osteomyelitis, metabolic disorders, or tumor excision [1]. Long bone defects is considered as a critical bone defect when they lose more than 50% of their diameter or more than 2 cm in length [2]. A wide variety of treatments have been attempted to treat critical bone defects. Autologous transplantation can also cause problems such as donor morbidity and mortality proportional to the size of the donated bone, limited availability of donor transplants, and generally low and unreliable rates of success [3].

The use of stem cells in the treatment of extensive bone defects has been studied extensively with high complexity with various outcomes with adipose tissue cord which are the most extensively studied [4].

Exosomes have been proven for the regenerative potential due to the functioning RNA components that could effectively stimulate tissue regeneration. The use of ASCs exosome is promising and has the potential to assist bone defects regeneration.

Platelet-rich fibrin (PRF) is also one of stem cell modality that has been used in previous studies. The ability of PRF to stimulate bone regeneration is due to the stimulating activity of growth factors released and activated platelets in proliferation of progenitor cells and vascularization at the wound site. Furthermore, PRF is relatively cheaper and easier to make compared to other biological modalities.

Despite several studies that used exosomes and PRF in treating bone defect, there has been no animal study that compared the use of ASCs exosome and PRF in long bone defects in mice. This study aimed to evaluate ASCs exosome and PRF implanatation in critical long bone defects Sprague-Dawley rats.

## Materials and methods

This study is a quasi-experimental post-test only control group test on experimental animals, Sprague-Dawley rats. Samples were randomly taken from male white Sprague-Dawley rats aged 8–2 weeks and weighed 250–350 g with no physical defects. We excluded rats with implant failure, infection around the area of operation, and mortality before termination.

These Sprague-Dawley rats were acclimatized for one week. A 5-mm femoral bone defect was created in each rat by osteotomy, followed by internal fixation with a 1.4-mm K-wire. An incision was made in the iliac wing to take the bone graft. The samples were then divided into five groups of six rats each, comprised of hydroxyapatite (HA) and bone graft treatment group (group I); HA, bone graft, and PRF (group II); HA, bone graft, and ASCs exosome 0.4 cc (group III); HA, bone graft, PRF, and ASCs exosome 0.4 cc (group IV); HA, PRF, and ASCs exosome (group V). After four weeks of observation, femur samples were taken for molecular analysis of BMP-2 and chordin levels and histomorphometry examination.

### PRF processing

Before the procedure, essential tools are UV-treated in a biological safety cabinet (BSC) for 30 min. After UV treatment, the materials are moved into the BSC. Fresh whole blood is pipetted into a 50-ml Falcon tube and labeled accordingly. Subsequently, 8 ml of this fresh whole blood is placed in a round-bottom tube labeled ‘PRP” and centrifuged at 3000 rpm for 5 min to obtain platelet-rich plasma (PRP). The top 2 ml of the supernatant is removed, and the remaining portion, typically 1–3 ml (preferably 2 ml), is retained. This supernatant is then transferred to a 15-ml Falcon tube, and 0.5 ml of 1 M CaCl_2_ is added to achieve a final CaCl_2_ concentration of 25 mM. If the PRP volume exceeds 2 ml, it is divided into a new 15-ml Falcon tube. This final product is labeled “PRF” and centrifuged at 1800×*g* for 57 min. The resulting PRF is collected and stored at room temperature in a general laboratory drawer for future use.

### Exosome processing

Cryotubes containing adipose-derived MSCs were thawed and cultured for 4 weeks, with conditioned medium (CM) collected at each medium change. To retrieve the CM, the frozen CM was thawed by immersing the container in warm water at 37 °C. It underwent a series of centrifugation steps at 750×*g*, 2000×*g*, and 10,000×*g* for 15 to 45 min, followed by filtration through a 0.2 µm syringe filter. The resulting filtrate was ultracentrifuged at 100,000×*g* for 90 min at 4 °C. The supernatant was discarded, and the pellet containing extracellular vesseles (EVs) was transferred to a 15-ml Falcon tube. Cold D-PBS was added to reach a volume of 5 ml, and the EVs were resuspended. They were divided into 1-ml aliquots, placed in cryovials, and stored at either − 20 °C or − 80 °C for up to 1 year. Additionally, Exo ASC preparation was done in D-PBS suspension and packaged in 1 cc syringes with a volume of 150 ul per syringe.

### Histomorphometry and PCR

Rat specimens were terminated at the fourth week using intraperitoneal Ketamine and Xylazine, followed by heart aspiration. Bone tissue was decalcified, and femur osteotomy was done with a 22-mm-diameter saw at callus proximal and distal parts. Tissues were fixed in 10% formalin for 24 h. Tissue preparation included dehydration in 70%, 80%, and 95% alcohol for 3 h each, followed by a clearing process in xylene solution for 3 h. The subsequent steps involved infiltration and embedding. The specimens were then observed under a light microscope and photographed using OptiLab at a 4× objective lens magnification with 3–7 fields of view to capture the overall microscopic image of the preparations. All microphotographs in the small field of view (40× magnification) were combined using the Stitching Fiji ImageJ plugin, deprecated, Stitch file in the directory (unknown configuration), and the stitched image matched the subject number.

For routine staining (HE) microphotographs, the measurement of the percentage of fibrosis + chronic inflammation and osseous tissue area was carried out, and comparisons were made among subjects within the groups using ImageJ. The percentage of area obtained through ImageJ was then presented in a graphical form and subjected to statistical testing

## Results

In this study, we found that none of the rats experienced mortality during the observation period, both before and after the procedure. In terms of subjective assessment of activity and eating patterns, the samples generally exhibited a favorable response. However, samples that underwent bone graft extraction showed slightly lower activity and eating patterns compared to samples that did not undergo bone graft extraction (group V).

On histomorphometric examination, the highest value for fibrosis area was found in group IV, with a value of 65.7 ± 7.9 (Table 1﻿). Meanwhile, for the percentage of supporting tissue area, the highest value was observed in group II of mice receiving HA, bone graft, and PRF, with a percentage area of 36.9 ± 12.7. The best results for void area in histomorphometry were found in group I of mice receiving HA and bone graft, with a value of 13.2 and a standard deviation (SD) of 9.1 (Tables 1, 2).

**Table 1:** Overall results from histomorphometry and PCR

Treatment groups	Average body weight (g)*	Histomorphometry area			Comparison of BMP-2 values between groups		Comparison of chordin levels between groups	
		Fibrosis area percentage (SD)	Osseous area percentage (SD)	Void area percentage (SD)	Median (Min–Max)	*p* value**	Median (Min–Max)	*p* value**
I: HA and Bone Graft (*n* = 6)	269 ± 20.1	53.7 (9.1)	33.1 (7.1)	13.2 (9.0)	1.1 (1.0–1.5)	0.336	1.0 (1.0–1.5)	0.9
II: HA, Bone Graft, and PRF (*n* = 6)	265.3 ± 20.8	57.827 (13.58)	37.0 (12.9)	6.0 (3.8)	0.5 (0.03–1.2)		1.4 (0.0–14.0)	
III: HA, Bone Graft, PRF, and ASCs exosome (*n* = 6)	227.3 ± 23.2	65.6 (66.1)	27.6 (5.0)	8.9 (6.2)	0.86 (0.04–2.67)		6.2 (0.0–117.4)	
IV: HA, Bone Graft, and ASCs exosome (*n* = 6)	237 ± 54.8	65.7 (7.9)	27.7 (8.4)	7.8 (5.6)	2.89 (0.09–13.00)		7.0 (0.02–51.3)	
V: HA, PRF, and ASCs exosome (*n* = 6)	259.7 ± 21.6	68.6 (7.5)	25.3 (7.1)	7.2 (3.6)	0.09 (0.04–3.1)		8.7 (0.01–73.5)

**Table 2:** The comparison between exosome and control group

	ASCs exosome + HA + BG (SD)/(Min–Max)	Control (SD)/(Min–Max)	Mean difference	*p* value
Osseous area	27.7 (8.4)	33.1 (7.1)	5.5	0.4*
Fibrosis area	65.7 (7.9)	53.7 (9.1)	− 12.0	0.4*
Void area	7.8 (5.6)	13.2 (9.0)	6.6	0.7*
BMP-2	2.9 (0.1–13.7)	1.1 (1.0–1.5)	–	1.0**
Chordin	6.2 (0.0–117.4)	1.0 (1.0–1.5)	–	1.0**

On molecular analysis, the BMP-2 value in group IV (HA, bone graft, and ASCs exosome) is the highest BMP expression among the research groups, followed by group I (HA and bone graft), group III (HA, bone graft, PRF, and ASCs exosome), group II, and group V, respectively. Chordin values in group V (HA, PRF, and ASCs exosome) showed the highest expression among the research groups, followed by group IV (HA, Bone Graft, ASCs exosome), group III (HA, bone graft, PRF, and ASCs exosome), group II, and group I.

The results showed that the group receiving PRF had a higher osseous area (37.0 ± 12.9) compared to the group receiving ASCs exosome (27.7 ± 8.4) (Table 3).

The administration of PRF in the research samples yielded better results compared to the control group. It appears that the osseous area in the PRF + HA + BG group (37.0 ± 12.9) was higher than in the control group (33.1 ± 7.1) (Table 4).

**Table 3:** The comparison between ASCs exosome + HA + BG and PRF + HA + BG group

	PRF + HA + BG (SD)/(Min–Max)	ASCs exosome + HA + BG (SD)/(Min–Max)	Mean difference	*p* value
Osseous area	37.0 (12.9)	27.7 (8.4)	9.2	0.7*
Fibrosis area	57.8 (13.6)	65.7 (7.9)	− 7.9	1.0*
Void area	6.0 (3.8)	7.8 (5.6)	1.4	1.0*
BMP-2	0.5 (0.03–1.2)	2.9 (0.1–13.7)	–	0.6**
Chordin	1.4 (0.0–14.0)	6.2 (0.0–117.4)	–	1.0**

**Table 4:** The comparison between PRF + HA + BG and control group

	PRF + HA + BG (SD)/(Min–Max)	Control (SD)/(Min–Max)	Mean difference	*p* value
Osseous area	37.0 (12.9)	33.1 (7.1)	3.8	1.00*
Fibrosis area	57.8 (13.6)	53.7 (9.1)	4	1.00*
Void area	6.0 (3.8)	13.2 (9.0)	− 8.0	0.3*
BMP-2	0.5 (0.03–1.2)	1.1 (1.0–1.5)	–	1.0**
Chordin	1.4 (0.0–14.0)	1.0 (1.0–1.5)	–	1.0**

**Fig. 1:**
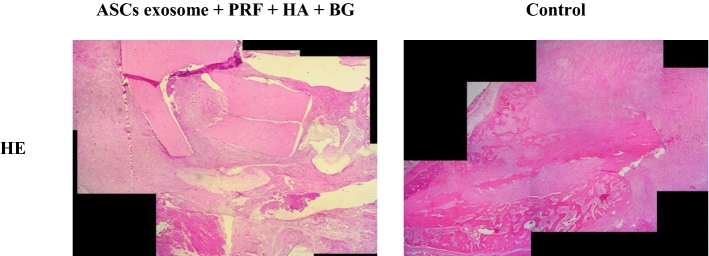
The results of histomorphometry examination of bone tissue in Sprague-Dawley rats between ASCs exosome + PRF + HA + BG and control group

**Fig. 2:**
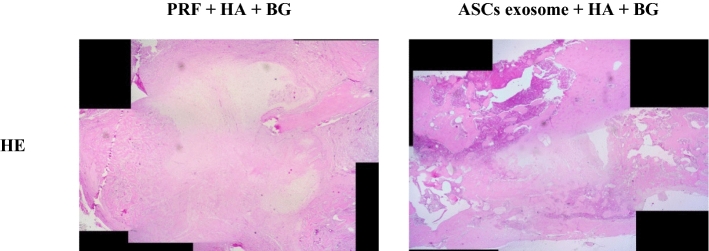
The results of histomorphometry examination of bone tissue in Sprague-Dawley rats between PRF + HA + BG and ASCs exosome + HA + BG group

From the analysis of the fibrosis area, it is evident that there was an increase in the group receiving a combination of ASCs exosome and PRF + HA + BG (65.6 ± 66.1) compared to the control group (53.7 ± 9.1). In contrast, in the osseous area, it appears that the control group had a greater increase (33.1 ± 9.1) compared to the group that received the combination treatment of ASCs exosome and PRF simultaneously (27.6 ± 5.0). (Figs. 1, 2).

## Discussion

The selection of experimental animals of Sprague-Dawley rats as models of critical bone defects is in accordance with previous studies considering its durability, birth turnover speed, and their tendency to be calmer in nature [5, 6]. We conducted a histomorphometry analysis by calculating the osseous area, fibrosis area and void area. It is in accordance with the stage of bone healing, fibrosis and chronic inflammation areas (granulation tissue) are forms of the description of the fibrovascular stage in the bone healing. ASCs exosome is able to increase the potential of osteogenic bone marrow mesenchymal stem cells (BMSCs) by activating the Wnt3a/*β*-catenin pathway and facilitating bone repair and regeneration abilities [7].

However, the osseous area in the control group (33.1 ± 7.1) was higher than in the treatment group (27.7 ± 8.4). This could be caused by the length of time the study was assessed that affects the soft callus growth process in the treatment group compared to the control group.

In this study, PRF administration, combined with HA and BG, showed good results compared to the control group. The osseous area in group II (37.0 ± 12.9) was higher compared to the control group (33.1 to ± 7.1). PRF administration can encourage bone regeneration by providing a scaffold for cell attachment and releasing growth factors that induce osteogenesis. PRF administration can increase the expression of Tnfrs11b mRNA encoding osteoprotegerin (OPG), encoding mRNA in osteoblast cells [7].

In this study, a comparison was made between the PRF administration group with ASCs exosome + HA + BG (group IV) on the callus growth process. It was found that the group II had a higher osseous area (37 ± 12.9) compared to group IV (27.7 ± 8.4). In terms of area of fibrosis and chronic inflammation (granulation tissue), group IV (65.7 ± 7.9) was higher than group II (57.8 ± 13.6). Unlike exosomes, PRF itself can be used as a single biological material or together with a graft to accelerate bone regeneration. PRF simultaneously plays a role in increasing angiogenesis, immunity, and epithelialization as the process of healing and maturation of soft tissues. A systematic study by Eid al-Haq et al. showed that PRF accelerated the healing process in rat bone defects compared to physiologically (without any intervention) [7].

In this study, an analysis of the effect of ASCs exosome was also carried out combined with treatment compared to the control group. From the results of the analysis of fibrosis area and granulation tissue appeared to increase in the group given the combination treatment of ASCs exosome, PRF, HA, and BG, or group III (65.6 ± 66.1) compared to the control group (53.7 ± 9.1). It is known that ASCs exosome has high osteogenic ability because they can trigger other progenitor cells and induce osteogenic differentiation. In a study conducted by Sart et al. that secretomes of adipose tissue origin can promote proliferation and migration through increased adhesion and cell proliferation [8].

To assess differences in bone growth, a comparison was made between the group given a combination treatment of ASCs exosome and PRF compared to the group given bone graft (group V). The fibrous area of the group III (68.6 ± 7.5) increased compared to group V (53.7 ± 9.1). However, based on Kruskal Wallis’ statistical test, there were no significant differences between the two research groups. In addition, it appears that BMP-2 and Chordin in the combination group of PRF and ASCs exosome are more increased compared to the bone graft group. It is known that BMP-2 is a protein that functions as a trigger for differentiation of mesenchymal stem cells (MSC) into osteoblasts by binding directly to bone morphogenic protein receptors (BMPR) on MSC, while Chordin is an adhesion inhibitor protein between BMP-2 and BMP receptors. This process occurs continuously and will affect the bone healing process.

Although the results obtained in this study did not show significant results, the equivalence of the use of ASCs exosome and PRF (group III) compared to controls using bone graft (group V). Bone graft and hydroxyapatite have been known as the main treatment in patients with bone defects, especially critical bone defects with good outcomes [9]. Previous research conducted by Pires et al. showed that the use of bone graft, and its substitution (hydroxyapatite) has a good outcome in overcoming critical bone defects [10]. However, the use of bone graft is associated with donor-site morbidity, which can impair the quality of life of patients. Such complications can include pain, infection, altered sensations, loss of tooth vitality, neurosensory disorders, and patient dissatisfaction [11].

In this study, the absence of significant differences showed that the use of PRF and ASCs exosome had an effectiveness that could be said to be equivalent to the use of a combination of bone graft and hydroxyapatite. In this study, it was found that there was production of osseus and fibrous tissue as soft callus which is equivalent to the use of bone graft. This can be useful in critical bone defect applications that no longer cause morbidity where donors can take bone grafts. With the development of science today, surgical approaches that are minimally invasive and do not cause further morbidity are the main choice. The lack of complications caused by donor sites for bone graft causes the application of ASCs exosome and PRF to be very extensive and significant. This study shows that the use of biological agents is feasible for the management of patients with critical bone defects.

## Limitations

There are several imitations of histomorphometry analysis in this study. Some micro photo stitching images are not perfect with overlays or imprecise micro-photo stitching and structure that can affect the subsequent percentage analysis of the area. Second, no special staining was made to mark the collagen extracellular matrix in the fibrosis area as with Masson Trichrome, or safranin O for the cartilage/bone matrix so that the image separation process based on the red, green, and blue channels was less precise in calculating the area of fibrosis, chronic inflammation, recurrence and void areas that wanted to be found in this study. Third, in determining the threshold when calculating the % area, there is a sliding scale that is placed manually so that it can be biased in determining the percentage of area. Fourth, determining the % void area using the formula 100% minus the known area, this also has the potential to cause bias.

For imitation of qRT-PCR BMP-2 in this study included limitations in almosthalf the number of subject’s not detected BMP-2 expression from samples of critical bone defect areas but triplication of BMP-2 expression was met for each group.

## Conclusion

The outcome of PRF combined with ASCs exosome without bone graft had no donor-site morbidity, in addition to histomorphometric and qRT-PCR results which were equivalent to other groups. The administration of ASCs exosome and PRF has a comparable outcome with the use of bone graft in terms of osseus area and expression of BMP-2 in critical bone defect.

## Data Availability

All the data are available can be accessed via corresponding email after clearly stating the intention and permission to conduct research that requires our data. We used SPSS for Windows version 25 for data analysis.
